# A comparative study of differences between parents and teachers in the evaluation of environmental sensitivity

**DOI:** 10.3389/fpsyg.2023.1291041

**Published:** 2023-12-20

**Authors:** Borja Costa-López, Rosario Ferrer-Cascales, Nicolás Ruiz-Robledillo, Natalia Albaladejo-Blázquez, Monika Baryła-Matejczuk

**Affiliations:** ^1^Department of Health Psychology, University of Alicante, Alicante, Spain; ^2^Institute of Psychology and Human Sciences, WSEI University, Lublin, Poland

**Keywords:** environmental sensitivity, highly sensitive child scale, temperament trait, children, parents, teachers

## Abstract

**Introduction:**

The inextricable bond between humans and the environment underscores the pivotal significance of environmental sensitivity. This innate trait encompasses a wide array of ways individuals perceive, process, and react to various internal and external stimuli. The evaluation of this trait in children is generally conducted by parents. However, little is known about the concordance of the parents reports with those conducted by others, such as teachers. Children’s behavior assessment is a current and relevant issue and finding out more positive results could make improvements in different contexts, such as home, clinics and schools.

**Objective:**

This study examines agreements and discrepancies between parents and teachers as raters of environmental sensitivity in Spanish children.

**Methods:**

Participants were 124 parents and eight teachers of youngsters between 3 and 10 years, who completed a paper survey providing information about parental and school variables and environmental sensitivity.

**Results:**

Parents and teachers mostly differ when rating environmental sensitivity, being parents the raters who score higher levels of this trait than teachers. Also, poor intra-class coefficients of reliability are found in both the items of HSCS, the dimensions and the general factor of environmental sensitivity among the informants.

**Conclusion:**

The present investigation provides novel findings related to inter-rater assessment on environmental sensitivity and how these different informants could affect in the report. This study also highlights the need of making and validating new and specific tools to assess environmental sensitivity for teachers.

## 1 Introduction

Environmental sensitivity (ES), under the theoretical meta-framework proposed by Pluess (2015), is described as the stable and heritable ability to process and interpret the information from both the external and internal stimuli. Recent authors have suggested sensitivity could show a continuum, in which individuals may present different levels of sensitivity (low, medium and high), due to the fact they could substantially differ in such responsivity to environment, as indicated from the *differential susceptibility theory* (Belsky, 1997; Belsky et al., 2007; Pluess, 2015).

For better or for worse, it seems that sensitivity could lead to positive and negative outcomes in the interaction with environment (Aron et al., 2012; Di Paola et al., 2022). Extensive evidence from past empirical studies and theoretical frameworks supports the notion that certain individuals have a heightened susceptibility to negative outcomes arising from adverse childhood experiences (Aron et al., 2012; Di Paola et al., 2022). Conversely, based on *vantage sensitivity theory* (Pluess and Belsky, 2013), these individuals tend to experience amplified advantages from positive influences within nurturing and supportive environments, owing to their heightened sensitivity (Aron et al., 2012; Di Paola et al., 2022).

In fact, from a personality and temperament perspective and according to the Environmental Sensitivity meta-framework, the variability of ES could be conceptualized as an individual trait (Aron and Aron, 1997; Pluess, 2015). Recent studies further suggest the significance of ES as a central overarching personality trait, with its diverse components found within numerous well-established psychological frameworks (Pluess, 2015). This psychological framework suggests that around 20–30% of people display heightened levels of sensory conscientiousness, behavioral inhibition, extensive cognitive processing of environmental stimuli, and significant emotional and physiological reactivity (Aron and Aron, 1997; Aron et al., 2012; Pluess, 2015; Lionetti et al., 2018; Pluess et al., 2018; Lionetti, 2020). Thanks to the hypothesis on neurosensitivity, the highly sensitive central nervous system even tends to lead an increased sensitivity and susceptibility to environmental events, and this allows to perceive and process experiences more deeply (Aron and Aron, 1997; Belsky and Pluess, 2009; Aron et al., 2012; Greven et al., 2019).

Measuring ES in children, recent studies have indicated a series of potential markers which have been detected at genetic, physiological, and psychological levels (Aron and Aron, 1997; Belsky et al., 1998; Boyce and Ellis, 2005; Belsky and Pluess, 2009; Pluess and Belsky, 2013; Sperati et al., 2022). Regarding the psychological level of analysis, the children’s ES can be measured by observational techniques or self-report questionnaires, which are popular because they appear to be relatively easy to administer, and to be potentially useful in clinical and health fields (Aron and Aron, 1997; Costa-López et al., 2022). The self-report version of the 12-item Highly Sensitive Child Scale (HSCS) is one of the most world-widely used scale (Weyn et al., 2021), and it presents not only an original UK validation, but also parent-report adaptations for Dutch (Slagt et al., 2018), Italian (Sperati et al., 2022), and Spanish (Costa-López et al., 2022) children. Extensive explorations of the HSCS in UK and other countries could also provide us with psychometric robust findings, which show a structure that captures a general sensitivity and three specific factors related to the perception of both external and internal stimuli (Lionetti, 2020): (1) ease of excitation, referring to heightened susceptibility to negative effects from a high level of activity in one’s surroundings; (2) esthetic sensitivity, which is the appreciation and sensitivity to positive stimuli, such as nice tastes and smells; and (3) low sensory thresholds, which are associated with the response to disturbing sensory contexts, such as loud noises or violent situations.

Considering the importance in the field of mental health of using multiple informants when assessing psychological aspects, there is however a lack of investigation on information obtained from teachers and parents as raters (Kazdin, 2005; Duvekot et al., 2015). In addition, dissimilar respondents frequently demonstrate a lack of agreement in their assessments of child behavior due to the varying environments in which parents and teachers observe the child, leading to differing viewpoints (van der Ende et al., 2012). Also, the *sensory processing sensitivity theory* may explain the difficulties in reaching agreements when evaluating ES in children, since it proposed that high sensitivity is featured by heightened behavior inhibition, emotional reactivity, sensitivity to subtle stimuli, and deeper cognitive processing to environmental stimuli (Aron et al., 2012). That means highly sensitive individuals usually demonstrate more internalizing behaviors than externalizing ones. In any case, as stated before, ES could be manifested in different ways depending on the context, and this could be a plausible mechanism to explain the possible differences that could be found between different raters in the assessment of ES.

In the realm of child assessment, families and educators assume vital roles as primary and diverse observers, offering valuable and distinct perspectives. Parents, in particular, hold significant importance as they serve as raters of their child’s behavior, owing to their extensive observations of the child’s actions a broad spectrum of situations, enabling a comprehensive understanding of the child’s growth and development process (Duvekot et al., 2015). Applied to educative contexts, for the assessment of some childhood psychological aspects, there is a need to take into account teachers’ ratings, as a complementary report of parents’ as well (Pelham et al., 2005). At schools, teachers have a valuable role since they can report children’s information regarding their daily social functioning and their interaction with other youngsters, as well as their performance (Pelham et al., 2005). Previous investigations on temperament have also highlighted that children’s self-reports usually differ from observers’ ones (Tackett, 2011; Luan et al., 2017). Indeed, self-report differs from parent and teacher ratings (Laidra et al., 2006; Barbaranelli et al., 2008; Göllner et al., 2017). Differences in personality traits become evident in the extent to which they are directly expressed through observable behaviors, leading to varying degrees of information provided to different perspectives (Vazire, 2010). In regard to the evaluation of personality and temperament traits, such as environmental sensitivity, they can potentially differ influencing what kind of information is identified and used (Vazire, 2010; Brandt et al., 2021). Adopting an informational approach, specific contexts are prone to elicit the manifestation of personality and temperament traits through observable behaviors, resulting in the varying visibility of certain traits compared to others (Tett and Burnett, 2003; Brandt et al., 2021). However, gaining different perspectives on children’s temperament by observing them in various situations enhances the depth and inclusiveness of their temperament description (Kraemer et al., 2003).

Regarding the increasing relevance of parent-teacher agreement in children’s psychological assessment, there is, however, a notable scarcity of research dedicated to this specific aspect (Major et al., 2015). Specifically, although the information obtained from teachers in the evaluation of psychological aspects is potentially valuable, in environmental sensitivity, to the best of our understanding, there is only two studies which have examined the teachers’ rating of this temperament trait in children. ES is therefore an under-researched trait. Moreover, as variations in individuals’ environmental sensitivity seem to anticipate diverse responses to the environment, ranging from behavioral issues to overall well-being, quality of life, and social competence, having both parent and teacher reports at hand enables the early detection of developmental alterations in children through a straightforward approach (Liss et al., 2005; Booth et al., 2015; Sobocko and Zelenski, 2015; Black and Kern, 2020; Costa-López et al., 2021; Yano et al., 2021). Additionally, it opens up possibilities for achieving beneficial outcomes by fostering supportive conditions (Liss et al., 2005; Booth et al., 2015; Sobocko and Zelenski, 2015; Black and Kern, 2020; Costa-López et al., 2021; Yano et al., 2021). The present study therefore aimed to investigate the screening of the parent and teacher-reported environmental sensitivity in children. We examined the comparison between parents and teachers in regards of the assessment of environmental sensitivity, not only the differences between these two informants, but also their relationship on the evaluation of this trait.

## 2 Methods

### 2.1 Study design

The present study employed a cross-sectional research approach to compare how parents and teachers rate environmental sensitivity in children.

### 2.2 Sample

According to our research objective, this study adopted random sampling technique to target specific people. Participants were recruited from kindergarten and primary educational centers, which were representative of the Spanish context. The selection process occurred between December 2020 and February 2021. Parents were recruited to fill out the instruments following these inclusion criteria: (a) individuals who were 18 years of age or older; (b) families with children attending a kindergarten or primary educational center; and (c) sufficient reading comprehension to complete the assessment protocol. Inclusion criteria for teachers were: (a) individuals who were 18 years of age or older; and (b) to be a teacher of a kindergarten or primary class in a Spanish educational institution (c) sufficient reading comprehension to complete the assessment protocol. Excluded from the study were parents and teachers who had sensory, physical, or psychological impairments that hindered their ability to comprehend and complete the evaluation instruments. Also excluded were parents and teachers of children diagnosed with any neurodevelopmental disorder, autism, or sensory modulation disorder.

A total of 124 families and eight teachers participated in this study. Parents’ mean age of 42.21 years (SD = 7.30), of whom 86.29% were women. The predominant educational level was the higher education both for mothers (43.55%) and fathers (40.18%). The children were aged between 3 and 10 years (*M* = 6.935; SD = 2.32), and 51.61% were boys (Table 1). The mean age of the teachers was 45.88 (SD = 9.67), and seven of them were women. Six of them got a degree in Education and two of them reached a master’s degree. All of them had full-time contracts.

**Table 1:** Sociodemographic data description of the sample (*N* = 124).

Parents	n/M (%/SD)	
	Women	Men
Age	42.04 (7.67)	43.29 (4.13)
Educational level		
Primary education	5 (4.59)	1 (6.67)
Secondary education	23 (21.10)	3 (20.00)
Vocational education	33 (15.42)	4 (30.28)
Higher education	48 (22.43)	7 (44.04)
**Teachers**	**n/M (%/SD)**	
	**Women**	**Men**
Age	46.57 (10.23)	41.00 (0.00)
Educational level		
Degree	5 (71.43)	1 (100.00)
Master’s degree	2 (28.58)	–
**Children**	**n/M (%/SD)**	
Gender		
Boys	64 (51.61)	
Girls	60 (48.39)	
Age	6.935 (2.32)	
Educational level		
Kindergarten (3–4 years old)	17 (13.71)	
Kindergarten (5–6 years old)	21 (16.94)	
Primary education (6–7 years old)	22 (17.75)	
Primary education (7–8 years old)	1 (0.81)	
Primary education (8–9 years old)	23 (18.55)	
Primary education (9–10 years old)	21 (16.94)	
Primary education (10–11 years old)	19 (15.32)	

### 2.3 Measures

For the collection of sociodemographic data, we designed an *ad hoc* questionnaire. The relevant information included in this study for the children comprised their age, gender, and educational level (kindergarten and primary school).

In evaluating the environmental sensitivity, we employed the Highly Sensitive Child Scale (HSCS; Pluess et al., 2018; Costa-López et al., 2022) developed and tested for its psychometric properties in the original version by Pluess et al. (2018). The assessment tool consisted of 12 items, which were further categorized into three subscales: (a) ease of excitation (EOE), (b) esthetic sensitivity (AES) and (c) low sensory threshold (LST). Participants responded to what extend the items described children on the basis of a 7-point Likert-type scale, ranging from (1) ‘Strongly disagree’ to (7) ‘Strongly agree’ (Muñiz et al., 2013). The HSCS has shown evidence of reliability and validity among parent-report children internal consistency of the Spanish HSCS total score was α = 0.84 and the HSCS subscales presented acceptable reliability scores with α = 0.86 for EOE, α = 0.78 for AES, and α = 0.73 for LST (Costa-López et al., 2022).

### 2.4 Procedure

This investigation has the approval of the Research Ethics Committee of the University of Alicante (UA-2022-05-23_2), and the Bioethics Committee of the University of Economics and Innovation in Lublin (16 December 2019), as taking part of a European project. To commence the study, we initiated contact with the principals of the education centers to present the primary objective of the research. Once the school management teams were briefed, we proceeded to meet with parents and teachers of students attending kindergarten and primary education levels. During these meetings, parents and teachers were informed about the goals and voluntary participation of the research. Parents and teachers who expressed interest in participating in the study and met the inclusion criteria provided their informed consent by signing the required documents. Subsequently, the researchers provided instructions to the participants on how to complete the questionnaires and addressed any inquiries or uncertainties they had. Furthermore, the parent (either mother or father) who spent more time with the child and had deeper understanding of the child’s behavior and temperament was designated to complete the questionnaire (Pluess, 2020).

### 2.5 Data analysis

All data were entered and analyzed in SPSS 28.0. First, we performed the reliability of the HSCS for parents and teachers for this study, being acceptable when internal consistency (α/ω) values were between 0.75 and 0.90, and considering it excellent with values over 0.90 (Koo and Li, 2016).

Then, we computed descriptive statistics in items, dimensions, and the general factor of the HSCS. Normality, independence, and homoscedasticity assumptions were performed. We conducted Student-T to test differences between parents and teachers’ report on children’s environmental sensitivity regarding the items, dimensions, and the general factor of the HSCS. The effect size was also calculated through Cohen’s δ. Typically, δ = 0.20 is considered small; δ = 0.50 a medium effect size; and δ = 0.80 a large effect size (Cohen, 1988). An analysis of covariance (ANCOVA) was performed to control the effect of the age and gender of the raters (parents and teachers) as covariate variables for the assessment of ES.

Intraclass correlation coefficients were run to examine the degree of agreement of the different informants. Values less than 0.50 are indicative of poor reliability, values between 0.50 and 0.75 indicate moderate reliability, values between 0.75 and 0.90 indicate good reliability, and values greater than 0.90 indicate excellent reliability (Koo and Li, 2016). The level of significance was set at *p* < 0.05 for all the data analysis.

Finally, we conducted Pearson’s and partial correlations between parents and teachers’ general and specific factors of the HSCS. For the interpretation, values from *r_xy_* = 0.00 to *r_xy_* = 0.10 are considered as null correlations; from *r_xy_* = 0.11 to *r_xy_* = 0.30, weak correlations; from *r_xy_* = 0.31 to *r_xy_* = 0.50, moderate correlations; and values from *r_xy_* = 0.51 to *r_xy_* = 1.00 show strong correlations (Hernández-Lalinde et al., 2018).

## 3 Results

### 3.1 Reliability of the parents and teachers’ highly sensitive child scale version

The overall internal consistency of the 12-item parents and teachers’ Spanish version of the HSCS was adequate (α/ω > 0.8). In regard to the dimensions, both the parents and teachers’ version indicated the highest reliability score (Table 2).

**Table 2:** Internal consistency of the parents and teachers’ HSCS version.

	Respondent	Cronbach’s alpha	McDonald’s omega	95% CI
EOE	Parents	0.854	0.861	0.808, 0.891
	Teachers	0.917	0.927	0.891, 0.938
LST	Parents	0.655	0.738	0.574, 0.724
	Teachers	0.664	0.810	0.547, 0.755
AES	Parents	0.738	0.783	0.680, 0.787
	Teachers	0.793	0.791	0.727, 0.847
HSCS	Parents	0.817	0.800	0.765, 0.861
	Teachers	0.830	0.837	0.782, 0.871

EOE, Ease of Excitation; LST, Low Sensory Threshold; AES, Aesthetic Sensitivity; HSCS, Highly Sensitive Child Scale; CI, Confidence Interval.

Most of the corrected item-total correlations for both the parents and teachers’ version were above 0.30, except for items 5 (Some music can make them really happy) and 7 (They do not like watching TV programs that have a lot of violence in them) in the parents’ HSCS, and items 1 (They notice when small things have changed in their environment), 5 (Some music can make them really happy) and 10 (They love nice tastes) in the teachers’ HSCS. It was observed that the reliability of the full scale improved slightly if items 5 and 7 for parents’ version were removed. The reliability of the scale also improved a bit if items 1, 5 and 10 for teachers’ version were removed (Table 3).

**Table 3:** Reliability characteristics of the parents and teachers’ Spanish version of the highly sensitive child scale.

	R_it_^c^		α-i		ω-i	
	Parents	Teachers	Parents	Teachers	Parents	Teachers
1	0.427	−0.008	0.807	0.855	0.800	0.869
2	0.593	0.724	0.791	0.796	0.786	0.798
3	0.406	0.378	0.808	0.825	0.801	0.849
4	0.566	0.671	0.794	0.800	0.783	0.787
5	0.237	0.225	0.819	0.836	0.809	0.856
6	0.604	0.716	0.791	0.797	0.793	0.798
7	0.245	0.314	0.828	0.829	0.821	0.846
8	0.700	0.714	0.780	0.797	0.774	0.790
9	0.491	0.522	0.801	0.815	0.782	0.821
10	0.399	0.296	0.809	0.831	0.802	0.855
11	0.507	0.702	0.800	0.798	0.800	0.803
12	0.451	0.574	0.805	0.810	0.790	0.803

r_it_^c^, correlation item-total test; α/ω-i, reliability if the item is dropped.

### 3.2 Differences between parent and teacher report when rating children’s environmental sensitivity

Regarding the differences between teachers and parents’ report when assessing high sensitivity in children, Table 4 shows that significant differences were found in the most of the HSCS items (except for items 2 and 4). As can be seen, parents reported significantly higher scores in all of the items, compared to teachers. Differences in items 1, 4, 6, 8, and 9 presented a small effect size (δ < 0.5). Also, a medium effect size was observed in items 3, 5, 7, 10, 11, and 12 (0.5 < δ < 0.8).

**Table 4:** Means, standard deviations, student T test/Mann–Whitney U-test, confidence intervals and effect sizes for the HSCS-12 items, dimensions and general factor.

	M (SD)		*t*	*p*	95% CI	δ
	Teachers (*n* = 124)	Parents (*n* = 124)				
HSCS 1. The child notices when small things have changed in their environment.	4.19 (1.650)	5.25 (1.507)	5.306	<0.001	[0.669, 1.460]	0.674
HSCS 2. Loud noises make them feel uncomfortable.	4.44 (1.351)	4.32 (1.868)	−0.584	0.560	[−0.529, 0.287]	−0.323
HSCS 3. The child love nice smells.	4.73 (1.504)	5.60 (1.324)	4.840	<0.001	[0.517, 1.225]	0.359
HSCS 4. The child gets nervous when they have to do a lot in little time.	4.58 (1.443)	4.37 (1.708)	−1.044	0.298	[−0.605, 0.186]	−0.382
HSCS 5. Some music can make them really happy.	3.36 (1.745)	5.79 (1.264)	12.543	<0.001	[2.046, 2.809]	1.306
HSCS 6. The child is annoyed when people try to get them to do too many things at one.	4.161 (1.334)	4.645 (1.472)	3.195	0.002	[0.241, 1.017]	0.154
HSCS 7. The child does not like watching TV programs that have a lot of violence in them.	3.35 (1.735)	4.75 (2.074)	5.745	<0.001	[0.917, 1.874]	0.472
HSCS 8. The child finds it unpleasant to have a lot going on at once.	3.20 (1.790)	4.45 (1.845)	5.415	<0.001	[0.795, 1.705]	0.431
HSCS 9. The child does not like it when things change in his/her life.	3.25 (1.709)	4.21 (1.717)	4.411	<0.001	[0.531, 1.388]	0.306
HSCS 10. The child loves nice tastes.	3.34 (1.753)	5.84 (1.340)	12.620	<0.001	[2.110, 2.890]	1.315
HSCS 11. The child does not like loud noises.	3.03 (1.437)	4.74 (1.771)	8.349	<0.001	[1.306, 2.113]	0.793
HSCS 12. When someone observes them, they get nervous. This makes them perform worse than normal.	3.08 (1.723)	4.08 (1.616)	4.714	<0.001	[0.582, 1.418]	0.344
Ease of Excitation	3.66 (1.07)	4.48 (1.19)	5.762	<0.001	[0.545, 1.110]	0.732
Low Sensory Threshold	3.61 (1.08)	4.76 (1.43)	7.150	<0.001	[0.834, 1.467]	0.908
Aesthetic Sensitivity	3.91 (1.00)	5.55 (0.83)	14.177	<0.001	[1.420, 1.878]	1.800
General Factor of Sensitivity	3.73 (0.95)	4.91 (0.86)	10.285	<0.001	[0.956, 1.409]	1.306

δ = Cohen’s d for the Student T-test’s effect size. CI, Confidence Interval.

In respect of the dimensions and the general factor of the HSCS, parents reported significantly higher scores than teachers. All of these differences showed a medium-large effect size (Table 4). Also, an ANCOVA was run to control the effects of age and gender of the raters for the assessment of ES. Despite finding a possible effect of the covariate variables, the significance of the initial effect of the rater does not disappear. Supplementary Table S1 presents the results of this analysis of covariance.

### 3.3 Inter-rater reliability with the intraclass correlation coefficient between parent and teacher report on children’s environmental sensitivity

Table 5 shows the intra-class coefficients for the reliability when reporting environmental sensitivity by parents and teachers. All the items of HSCS, the dimensions and the general factor of sensitivity showed poor reliability between parents and teachers reports on environmental sensitivity (ICC < 0.5).

**Table 5:** Inter-rater reliability correlations between parents and teachers in HSCS-12 items, dimensions and general factor.

	ICC	95% CI
HSCS 1	−0.038	−0.481, 0.272
HSCS 2	0.162	−0.195, 0.413
HSCS 3	−0.192	−0.700, 0.164
HSCS 4	0.360	0.088, 0.552
HSCS 5	−0.078	−0.538, 0.244
HSCS 6	0.407	0.154, 0.584
HSCS 7	0.193	−0.151, 0.434
HSCS 8	0.411	0.160, 0.587
HSCS 9	0.299	0.000, 0.508
HSCS 10	0.100	−0.283, 0.369
HSCS 11	0.167	−0.189, 0.461
HSCS 12	0.431	0.188, 0.601
EOE	0.195	−0.148, 0.436
LST	0.096	−0.289, 0.367
AES	−0.051	−0.499, 0.263
HSCS general factor	−0.057	−0.345, 0.339

ICC, Intra-class coefficients; CI, Confidence intervals; EOE, Ease of Excitation; LST, Low Sensory Threshold; AES, Aesthetic Sensitivity; HSCS, Highly Sensitive Child Scale.

### 3.4 Relationship between parents and teachers’ report on children’s environmental sensitivity

Table 6 shows Pearson correlations among the dimensions and the general factor of both the parents and teachers HSCS version. Strong correlations were found between dimensions and general factor of sensitivity among teachers. The correlation matrix showed weak/moderate associations between EOE, LST, and HSCS general reported by teachers and EOE dimension of parents’ version. Also, LST correlated moderately with EOE dimension of the parents’ version. HSCS general factor reported by parents demonstrated strong correlations with its dimensions, and weak correlations with EOE and LST dimensions reported by teachers.

**Table 6:** Pearson’s correlations and confidence intervals between parents and teachers’ report on environmental sensitivity in children.

	1	2	3	4	5	6	7	8
1. EOE (teachers’ versión)	–							
2. LST (teachers’ versión)	0.706*** [0.605, 0.785]	–						
3. AES (teachers’ versión)	−0.098 [−0.269, 0.080]	0.226* [0.052, 0.387]	–					
4. HSCS general factor (teachers’ version)	0.835*** [0.772, 0.882]	0.877*** [0.828, 0.912]	0.424*** [0.267, 0.558]	–				
5. EOE (parents’ versión)	0.298** [0.128, 0.450]	0.319*** [0.151, 0.469]	−0.084 [0.094, −0.256]	0.262** [0.090, 0.419]	–			
6. LST (parents’ versión)	0.095 [−0.083, 0.267]	0.073 [−0.105, 0.246]	−0.054 [−0.228, 0.123]	0.062 [−0.115, 0.236]	0.368*** [0.205, 0.511]	–		
7. AES (parents’ versión)	−0.029 [−0.204, 0.148]	0.083 [−0.094, 0.119]	−0.059 [−0.232, 0.119]	−0.015 [−0.191, 0.161]	0.246** [0.073, 0.405]	0.244** [0.071, 0.403]	–	
8. HSCS general factor (parents’ version)	0.205* [0.029, 0.368]	0.249** [0.076, 0.408]	−0.093 [−0.265, 0.085]	0.175 [−9.71×10^−4^, 0.341]	0.833*** [0.769, 0.880]	0.707*** [0.606, 0.785]	0.609*** [0.485, 0.709]	–

*p < 0.05, **p < 0.01, ***p < 0.001, EOE, Ease of Excitation; LST, Low Sensory Threshold; AES, Aesthetic Sensitivity; HSCS, Highly Sensitive Child Scale.

Pearson’s partial correlations were also performed for controlling the effect of age and gender of the raters. Same pattern of correlations was found compared to the initial ones (See Supplementary Table S2).

## 4 Discussion

The main aim of this research was to compare parental and teacher reports in assessing children’s environmental sensitivity.

Based on our findings on the reliability of the HSCS for the use in teachers and parents, values could show a reliable and an accurate instrument for assessing ES in children. These results are coherent with the previous research in which the validation of the instrument has been conducted with parents (Pluess et al., 2018; Costa-López et al., 2022) Moreover, in the case of teachers, results are also consistent with previous studies (Tillmann et al., 2018; Lionetti et al., 2021). In this sense, Tillmann et al. (2018) successfully adapted and validated a teacher-report German version of the HSCS as well. These researchers demonstrated good values in validity and a well-adjusted confirmatory factor structure of the test, including aspects related to children’s performance. Thanks to this, they could investigate on other educative variables which may influence on the development of the youngsters. In addition, Lionetti et al. (2021) developed a 17-item teacher-report measure for sensitivity in primary schoolers from Switzerland and Italy. They found this instrument perfectly captured features of sensitivity in school context.

We then tested whether assessment on children’s environmental sensitivity differs between parents and teachers’ report. As can be seen, when reporting children’s environmental sensitivity, parents and teachers could differ significantly in most of the items and all the dimensions of this temperament trait (*p* < 0.001). This is in line with the results of numerous prior cross-sectional studies, emphasizing the substantial differences among assessors in the information they consider while making personality and temperament judgments (Connelly and Ones, 2010; Vazire, 2010). Comprehending a person fully does not rely on a single perspective, as both self-awareness and insights from others contribute essential information (Vazire and Mehl, 2008). To gain a comprehensive understanding of developing individuals, it is necessary to involve multiple informants to gather diverse perspectives (Luan et al., 2017). Moreover, as parents and teachers could interact with children in different contexts, they may have access to a great variety of behavioral features (Major et al., 2015; Brandt et al., 2021). Environmental sensitivity as a temperamental trait depends on the characteristics of specific contexts (schools, home, clinics), so its expression leads to more relevant information related to children’s temperament (Major et al., 2015; Ramsey et al., 2016). In light of these findings, it is also justifiable to assume that teachers might possess valuable insights into temperamental traits that impact children’s performance (Brandt et al., 2021). Schools are contexts which demand these traits and that is the reason performance associated with temperamental traits is more reflected in children’s behaviors (Lechner et al., 2017). According to these results, parents also could report higher scores when rating children’s environmental sensitivity than teachers. These findings are coherent to previous studies, since parents seem to report their children as showing greater variety of behaviors than teachers do (Strickland et al., 2012; Major et al., 2015). Parents often occupy a privileged position to offer unique insights into the child’s life, challenges, and external factors that can influence their behavior (Major et al., 2015; Brandt et al., 2021). Thus, those two items of the HSCS (items 2 and 4) in which they agree, they describe external and observable behaviors that could be easier to be rated by different raters. Indeed, studies in the research literature have shown that parents and teachers typically exhibit stronger agreement when it comes to externalizing behaviors, while internalizing behaviors, being less observable, may lead to less consensus between them (Gagnon et al., 2007).

In regard of the inter-rater reliability obtained, the findings also demonstrated that parents and teachers seemed to not agree in all the dimensions of environmental sensitivity, and they appeared to not reach a good agreement for the general factor of this temperament trait. This goes without saying that discrepancies are usual between parents and teachers. Although teachers could see many behaviors of the children at school, parents spend much time with them, and they could observe a great variety of behaviors. Teachers usually have a group of children, and these raters need to pay attention to specific ES features. They may be able to capture school characteristics, such as child performance or social interactions, but it struggles with cognitive or emotional aspects. This could explain the agreement difficulties when assessing deeper psychological variables. Based on the literature, inter-rater reports of different informants have tended to be weak or moderate so far (Gagnon et al., 2007; Major et al., 2015; Brandt et al., 2021). Therefore, parent-teacher agreement matters. If it is not reached, it cannot be well understood. Previous researchers have suggested that ratings of children’s behavior by different close informants, such as parents and teachers, can be influenced by individual differences among sources caused by factors as stress or just personality and temperament differences or empathy that can influence the findings (Major et al., 2015).

Pearson correlations also pointed out the highly importance of making new instruments on environmental sensitivity in school contexts. Weak/moderate correlations of EOE dimension between parents and teachers’ version demonstrated that teachers could report external and social behaviors, and the most observable part of environmental sensitivity in children (Pluess et al., 2018). Poor correlations in heightened sensitivity between teachers and parents report actually demonstrate the need of investigation in schools and also indicate the lack of the specific tools for education professionals (Gabbert, 2023). Research is being conducted in this direction and the first results indicate the specificity of assessing a child’s behavior in the school environment related to the possibility of observation and attention to core sensitivity (Lionetti et al., 2021). Previous research suggests that developing environmental sensitivity tools for teachers may help to educate them about this personality and temperament trait, and also to examine the effects of high sensitivity on school performance, well-being and psychological adjustment (Greven et al., 2019). Also, poor correlations would not necessarily mean that the instrument is not reliable or valid. In fact, for this study the tool shows good values for psychometric properties. Weak/poor correlations could indicate that the instrument has a lack of context-related items, which is extremely valuable when evaluating ES by different observers.

There are certain limitations to this study. The main limitation pertains to the applicability of the findings to clinical populations due to the recruitment of participants from a community sample of individuals without specific clinical conditions. Second, the size of this sample is small, and results have to be interpreted with caution. Third, it is important to acknowledge that a segment of the data for this study was acquired through teacher reports, which necessitates careful consideration while interpreting the results, given the potential variability in the validity of these observers’ reports. In order to obtain more accurate information of environmental sensitivity, in future studies it would be useful to include other self-report measures. Fourth, there is a lack of research about environmental sensitivity that includes different raters, which can limit the discussion and conclusions of the results.

Despite the advantages that an instrument like HSCS may have to detect high sensitivity in children in schools, there is only one teacher version so far (Lionetti et al., 2021), so it would be interesting to validate the scale with larger samples and to replicate it in other countries. Indeed, educative institutions are essential in children’s lives and to consider in the way that they differ substantially in their sensitivity to the environment (Tillmann et al., 2018). Children spend most of their time at schools, where they learn how to face a great variety of experiences for their development. Additionally, creating new questionnaires for identifying ES characteristics in children, which includes educative aspects, may help researchers to link this temperamental trait to other school variables (Tillmann et al., 2018). Therefore, more reliable and valid tools for assessing ES should be developed to make children’s needs available. Also, assessment tools for parents and teachers should be different since the environment is diverse. It is crucial to create specific tools for each observer to specifically capture environmental sensitivity features in several contexts, and to gain an accurate profile of this temperamental trait by assessing ES in children in different fields of their lives.

Moreover, our results highlight the relevance of considering all the raters’ perspectives. They could add value in predicting behaviors in children. For instance, social manifestations of personality and temperament traits seem to be highly relevant for explaining variance in children’s performance at school. This illustrates the diversity in how personality and temperament are expressed across various contexts and the potential influence these variations may have on how different observers perceive and interpret personality and temperament (Brandt et al., 2021).

The findings of this study may provide valuable insights for both theory and practice in different contexts, including health and education. Despite the study limitations, this investigation has important implications for the psychological assessment and, specifically, for environmental sensitivity as a personality and temperamental trait. Our findings widely support the combination of different reporters, which appears to be recommended in research, in order to capture children’s profiles, since each rater could capture something unique (Brandt et al., 2021; Matlasz et al., 2023). However, the need of further research is important to explore the reliability between parent-teacher and self-report. Also, our results highlight the importance of taking into account children’s trait characteristics to detect early difficulties related to their well-being and quality of life. Health and education professionals may pay attention to that environmental sensitivity trait, especially, those highly sensitive children who could benefit from prevention and intervention programs (Pluess and Boniwell, 2015).

## Data availability statement

The original contributions presented in the study are included in the article/Supplementary material, further inquiries can be directed to the corresponding author.

## Ethics statement

The studies involving humans were approved by University of Alicante and the Bioethics Committee of the University of Economics and Innovation in Lublin. The studies were conducted in accordance with the local legislation and institutional requirements. The participants provided their written informed consent to participate in this study.

## Author contributions

BC-L: Conceptualization, Data curation, Formal analysis, Investigation, Methodology, Software, Writing – original draft. RF-C: Conceptualization, Investigation, Methodology, Supervision, Visualization, Writing – review & editing, Funding acquisition, Project administration, Resources. NR-R: Conceptualization, Investigation, Methodology, Supervision, Visualization, Writing – review & editing. NA-B: Investigation, Supervision, Writing – review & editing, Conceptualization, Methodology. MB-M: Funding acquisition, Investigation, Project administration, Resources, Supervision, Validation, Visualization, Writing – review & editing.
