# Supplementary material for: A comparative study of differences between parents and teachers in the evaluation of environmental sensitivity
Source: Front Psychol. 2023 Dec 20;14:1291041. doi: 10.3389/fpsyg.2023.1291041 (PMC10771387; doi:10.3389/fpsyg.2023.1291041)
Supplement: Supplementary file 1 [file Table_1.docx]

SUPPLEMENTARY MATERIAL

Table S1. Analysis of covariance for the assessment of environmental sensitivity in children with age and gender of the raters as covariate variables

| **Items** |  | **F_1, 244_** | **p** | **η^2^** |
| --- | --- | --- | --- | --- |
| HSCS 1. | Rater | 26.13 | <0.001 | 0.097 |
|  | Gender | 0.06 | 0.814 | 2.044e-4 |
|  | Age | 0.32 | 0.571 | 0.001 |
| HSCS 2. | Rater | 0.15 | 0.700 | 5.989e -4 |
|  | Gender | 1.56 | 0.214 | 0.006 |
|  | Age | 2.38 | 0.124 | 0.010 |
| HSCS 3. | Rater | 21.91 | <0.001 | 0.082 |
|  | Gender | 1.68 | 0.197 | 0.006 |
|  | Age | 0.03 | 0.859 | 1.177e -4 |
| HSCS 4. | Rater | 1.14 | 0.287 | 0.005 |
|  | Gender | 3.39 | 0.067 | 0.014 |
|  | Age | 0.10 | 0.753 | 3.991e -4 |
| HSCS 5. | Rater | 176.78 | <0.001 | 0.405 |
|  | Gender | 9.89 | 0.002 | 0.023 |
|  | Age | 6.33 | 0.013 | 0.014 |
| HSCS 6. | Rater | 13.72 | <0.001 | 0.050 |
|  | Gender | 3.53 | 0.061 | 0.013 |
|  | Age | 11.89 | <0.001 | 0.044 |
| HSCS 7. | Rater | 36.07 | <0.001 | 0.125 |
|  | Gender | 6.69 | 0.010 | 0.023 |
|  | Age | 0.90 | 0.343 | 0.003 |
| HSCS 8. | Rater | 39.63 | <0.001 | 0.130 |
|  | Gender | 0.04 | 0.840 | 1.332e -4 |
|  | Age | 21.67 | <0.001 | 0.071 |
| HSCS 9. | Rater | 28.16 | <0.001 | 0.095 |
|  | Gender | 0.06 | 0.810 | 1.957e -4 |
|  | Age | 23.28 | <0.001 | 0.079 |
| HSCS 10. | Rater | 194.83 | <0.001 | 0.418 |
|  | Gender | 1.50 | 0.221 | 0.003 |
|  | Age | 25.58 | <0.001 | 0.055 |
| HSCS 11. | Rater | 74.33 | <0.001 | 0.230 |
|  | Gender | 2.81 | 0.095 | 0.009 |
|  | Age | 2.43 | 0.121 | 0.007 |
| HSCS 12. | Rater | 26.83 | <0.001 | 0.096 |
|  | Gender | 5.73 | 0.017 | 0.020 |
|  | Age | 4.38 | 0.037 | 0.016 |
| Ease of Excitation | Rater | 29.13 | <0.001 | 0.100 |
|  | Gender | 0.19 | 0.667 | 6.370e -4 |
|  | Age | 17.92 | <0.001 | 0.062 |
| Low Sensory Threshold | Rater | 38.64 | <0.001 | 0.007 |
|  | Gender | 3.42 | 1.98 | 0.007 |
|  | Age | 2.97 | 0.086 | 0.010 |
| Aesthetic Sensitivity | Rater | 185.33 | <0.001 | 0.424 |
|  | Gender | 1.11 | 0.294 | 0.003 |
|  | Age | 6.19 | 0.014 | 0.014 |
| General Factor of Sensitivity | Rater | 100.58 | <0.001 | 0.280 |
|  | Gender | 0.42 | 0.516 | 0.001 |
|  | Age | 14.26 | <0.001 | 0.040 |

**Note**. η^2^= Eta Squared. ANCOVA-test’s effect size.
