# Supplementary material for: A comparative study of differences between parents and teachers in the evaluation of environmental sensitivity
Source: Front Psychol. 2023 Dec 20;14:1291041. doi: 10.3389/fpsyg.2023.1291041 (PMC10771387; doi:10.3389/fpsyg.2023.1291041)
Supplement: Supplementary file 2 [file Table_2.docx]

SUPPLEMENTARY MATERIAL

Table S2. Pearson’s partial correlations between parents and teachers’ report on environmental sensitivity in children, controlling gender and age of the raters.

|  | 1 | 2 | 3 | 4 | 5 | 6 | 7 | 8 |
| --- | --- | --- | --- | --- | --- | --- | --- | --- |
| 1. EOE (teachers’ version) | - |  |  |  |  |  |  |  |
| 2. LST (teachers’ version) | 0.706*** | - |  |  |  |  |  |  |
| 3. AES (teachers’ version) | -0.086 | 0.235** | - |  |  |  |  |  |
| 4. HSCS general factor (teachers’ version) | 0.837*** | 0.877*** | 0.430*** | - |  |  |  |  |
| 5. EOE (parents’ version) | 0.286** | 0.319*** | -0.049 | 0.268** | - |  |  |  |
| 6. LST (parents’ version) | 0.087 | 0.069 | -0.043 | 0.061 | 0.360*** | - |  |  |
| 7. AES (parents’ version) | -0.045 | 0.077 | -0.037 | 0.018 | 0.218** | 0.234** | - |  |
| 8. HSCS general factor (parents’ version) | 0.189* | 0.246** | -0.060 | 0.177 | 0.823*** | 0.709*** | 0.596*** | - |

**Note**. *p<0.05, **p<0.01, ***p<0.001, EOE = Ease of Excitation, LST = Low Sensory Threshold, AES =Aesthetic Sensitivity, HSCS = Highly Sensitive Child Scale.
